# Repeated Evaluations of Testes and Semen Characteristics in Two Binturongs (*Arctictis binturong*)

**DOI:** 10.3389/fvets.2021.658573

**Published:** 2021-03-12

**Authors:** Zainal Zahari Zainuddin, Symphorosa Sipangkui, Mohd Farqhan Kelana, Yap Keng Chee, Mohamed Reza Mohamed Tarmizi, Pierre Comizzoli

**Affiliations:** ^1^Borneo Rhino Alliance, c/o Faculty of Science and Natural Resources, Universiti Malaysia Sabah, Kota Kinabalu, Malaysia; ^2^Sabah Wildlife Department, Kota Kinabalu, Malaysia; ^3^Smithsonian Conservation Biology Institute, National Zoological Park, Washington, DC, United States

**Keywords:** binturong, testis measurements, semen evaluation, seasonality, sperm abnormalities, sperm cryopreservation

## Abstract

The binturong is a medium size carnivore belonging to the Viverrid family that lives in dense forests of South-East Asia. In addition to the protection of this vulnerable species in its natural habitat (*in situ*), conservation breeding efforts (*ex situ*) aim at maintaining a good genetic diversity while increasing the number of individuals to reinforce wild populations. Both approaches require a solid understanding of binturong's basic biology. However, there is still a lack of precise information about reproduction. The objective of this brief research report was to analyze testicular sizes and semen characteristics at different times of the year to better understand the reproductive physiology and inform future conservation efforts. A secondary objective was to describe sperm cryotolerance for the first time in that species. Examinations of testes and semen collections were conducted on two adult males. While testicular measurements were relatively constant across multiple examinations, semen characteristics (volume, viability, sperm concentrations, sperm motility) varied between samples. However, incidence of sperm morphological abnormalities was consistently high. Sperm cryotolerance appeared to be poor but further studies are warranted. The present dataset will be useful for future research on binturong reproduction and for the development of assisted reproductive techniques and biobanking of germplasms in that species.

## Introduction

The binturong is a medium size carnivore belonging to the Viverrid family, living in dense forests of South-East Asia (1, 2). Nine different subspecies vary little and can be easily distinguished by their size or geographical origin (3, 4). Mainly because of the loss of natural habitat, the status of the wild population across its native range is classified as Vulnerable by the International Union for the Conservation of Nature (5). In addition to *in situ* conservation, *ex situ* efforts in zoos and breeding centers are crucial to protect species for the long term (6, 7). However, both approaches require a solid understanding of binturong's basic biology to design adapted conservation strategies. This will ensure a good genetic diversity while increasing the number of individuals to reinforce wild populations (3, 4, 8). Knowledge about binturong physiology is still limited to few studies (9, 10). There is still a lack of precise information about reproduction, which limits subsequent development of assisted reproductive techniques that could enhance breeding efforts (8). Natural mating has been observed all year round but births tend to occur more often between February and April and then later in July and November after a gestation period lasting about 3 months (1, 10). Binturong can live past the age of 20 years in captivity, but the exact length of their reproductive life is not known. Early studies have reported that males can be proven breeders until the age of 15 years (1). Preliminary data on sperm lengths showed that characteristics were not very different from other carnivores, but more morphological details are still needed (11). Similarly, testicular measurements and semen quality have not been analyzed thoroughly. Lastly, semen cryopreservation has not been reported in that species.

Using a systematic approach like in similar reports on Sunda clouded leopards (12) and Malayan pangolins (13), the objective of the study was to analyze testicular measurements and semen characteristics at different times of the year to better understand the reproductive biology and inform future conservation efforts. A secondary objective was to assess sperm cryotolerance for the first time in that species.

## Materials and Methods

### Animals and Husbandry

The study was conducted on two wild-caught, adult male binturongs (Binturongs 1 and 2), with institutional approval from Sabah Wildlife Department (*via* letter with reference number JHL (HQ)400-9/82Jld.9(9)). At the time of examinations, animals were 12–14 year old. These were the only captive individuals in Sabah, on the island of Borneo (a third one was available but too old and overweight for the study). Animals were housed in separate, raised floor enclosures at Lok Kawi Wildlife Park, Kota Kinabalu, Sabah. Both binturongs were paired with females. Cages measured 3 × 1.5 × 1.5 m (length, width and height, respectively). Animals were fed once a day, consisting of a mixture of fruits (banana, papaya) and occasional chicken meat. Clean water was available *ad libitum*. Body weights were measured during each anesthesia, prior to electroejaculation (see below). Mean weights ± SD for Binturongs 1 and 2 were 10.2 ± 1.2 kg and 11.6 ± 0.9 kg, respectively.

### Anesthesia

The original plan was to collect individuals once a month to precisely document annual variations and explore the possibility to collect semen for systematic cryo-banking at any time of the year. However, several scheduled procedures had to be canceled due to logistical issues. A total of 11 procedures were conducted in Binturong 1 and 5 procedures in Binturong 2 at random times of the year between May 2018 and August 2020 (see Supplementary Material for details). Animals were fasted 12–18 h before drug administration and kept in individual cages. Each male received an induction dose of 0.05 mg/kg Medetomidine (Medetomidine 1 mg/mL, SedaMed, Ceva, France), 10 mg/kg ketamine-HCl (ilium ketamil 100 mg/mL, Troy Laboratories, Australia) and 0.5 mg/kg Butorphanol (Butorphanol 50 mg/mL, Kyron, South Africa) administered intramuscularly (IM) *via* blowpipe system (Telinject, GmbH, Germany). Once the individual was approachable, body weight was recorded using a digital weighing scale. Blood oxygen saturation (SpO_2_) was monitored using a pulse oximeter (EDAN, USA) with the probe clipped to the tongue. Body temperature was checked using a hand-held digital thermometer. Anesthetic depth was assessed by jaw tone, anal tone, palpebral reflex, pupillary light reflex, and response to noxious stimulus (withdraw-al reflex). An intravenous (IV) port was placed for anesthetic drug top-up and for fluid supplementation at a rate of 10 mL/kg/h. Once general anesthesia was reached, animals were transferred to an examination table for semen collection. After completion of all procedures, animals were moved back to a holding box. An antagonistic drug Atipamezole (Alzane 5 mg/mL, Zoetis, Spain) was administered IM at a dose five times the dose of medetomidine. Animals recovered undisturbed.

### Examinations of Reproductive Tracts

Examination was carried out with the binturong on right lateral recumbency. Once the fur was clipped around the scrotum and prepuce, the site was thoroughly cleaned using a saline solution and then wiped. Examination of the testicles and penis was carried out for external lesions. Both testicles were palpated for consistency, mobility in the scrotum and symmetry. Testicular consistency score was based on firmness (how much the testicular tissue could be depressed) and resilience (ability of the testicular tissue to return to normal shape after pressing). Scores were (1) very firm, (2) firm, (3) moderate, (4) soft, and (5) very soft. Both testes were also measured for length (L; cranial—caudal), width (W; medial—lateral), and height (H; dorso-ventral), using ultrasonography (Sonosite M-Turbo®, USA with an L52 × linear probe, 10–5 MHz transducer). Subsequently, the total testicular circumference was calculated. Each testicular volume was calculated, using a formula, L × W × H × 0.711. The total testicular volume was determined by combining the volume of the left and right testes like in previous reports (12). Subsequently, a transrectal ultrasound was performed to visualize and take measurements of the prostatic lobe.

### Electroejaculation

Prior to the procedure, the rectum was emptied of fecal materials and well-lubricated with non–spermicidal gel (K- Y Jelly, Johnson & Johnson Co., Arlington Tx, USA). The genital region was thoroughly cleaned with saline and then wiped with paper towel or gauze. The prepuce was retracted with the thumb and index finger and the penis cleaned. Once the penis was secured inside a 15-ml conical graduated tube, the prostate gland was palpated to determine the depth of rectal probe insertion (~5–8 cm). Stimulations were performed using an electroejaculator with a standard rectal probe measuring 2.2 × 8 cm (diameter and length), consisting of three 4 × 5.5 mm electrodes (Seager®, Dalzell Medical Systems, USA). The rectal probe was lubricated and gently pushed into the rectum close to the prostate glands (with ventral position of the electrodes). In general, the stimulation consisted of 2–3 series of 2–5 volts. On two occasions, the voltage was increased to 7 volts (Supplementary Material). Each series was divided into 2–3 sets of 10 stimuli each and gradually increased to the maximum. Each stimulus lasted about 3 s, followed by 4–5 s of rest before the next stimulus. The quality of stimulations was also verified based on the response of the hind leg contractions and penile erection. Occasionally, rectal mas-sage was used at the start or in-between stimulation, to flush the semen.

### Semen Handling and Evaluation

The semen evaluation followed our standard protocol for carnivores (12). Each fraction of semen ejaculated between stimulations was collected in a 15-ml tube and was examined for appearance, pH, volume, sperm concentration, viability, and morphology. Urine contamination were initially suspected based on the color (yellowish) and consistency (watery) of the semen sample. pH strips (Whatman International Ltd, Maidstone, UK) were used as an indicator of urine contamination (with reading <7). Semen was immediately transferred into a centrifuge tube and into a water-bath, at 37.5°C. The total semen volume was recorded at the end of the electroejaculation. A volume of 3–5 μl of raw, un-diluted semen was transferred onto a glass slide, covered with a cover-slip and examined under a phase contrast microscope (Olympus CX43-32P01, Japan) at a 200 × magnification, for assessing motility and progressive motility. Sperm vigor was evaluated on a scale of 0–5 (from 0% motility to 90% of active motile sperm) (12). Sperm motility was expressed as % of cells actively moving in a forward movement. The forward progressive sperm motility (%) was measured by placing 10 μl of semen on a prewarmed (37°C) glass slide with a coverslip and 200 sperm cells were counted. All fractions of ejaculate with sperm vigor of ≥2 were pooled together. To assess sperm concentration, an aliquot of 2 μl semen mixture in 38 μl of 4% formalin in saline with a dilution factor of 20 × was uploaded in a haemocytometer and examined under 400 × magnification. Using the sperm motility (%) and the progressive motility, a Sperm Motility Index was calculated using a standard formula (12).

To count the live vs. dead sperm cells, a ratio of 1 part semen to 3 parts Eosin-Nigrosin was chosen to avoid too high concentration of sperm on the thin smear, A similar ratio of semen were processed to Diff-Quick stain for morphological examination. Acrosomal integrity was evaluated using a single stain method. Small volume of semen (1 μl) was diluted with 9 μl of 2.9% sodium citrate dihydrate. Staining solution (10 μl) was added to the diluted semen and incubated for 70 s at room temperature. Part of the mixture (10 μl) was used to prepare a smear on a glass slide and examined under 1000 × on a phase contrast microscope. A minimum of 200 spermatozoa were examined for each semen sample. Morphological defects observed in spermatozoa were classified as primary (disorders during spermatogenesis) or secondary (after spermiogenesis or during semen handling). The primary sperm abnormalities included abnormal heads, nuclear vacuoles, and structural abnormalities of midpiece or tail. Secondary abnormalities detached heads, simple bent tails and loosen or detached acrosomes (14).

Semen fractions (sub-samples collected during different stimulations) of similar quality were pooled in a 2-ml vial and mix with Tris/Ham F-10 (Gibco/Thermofisher, USA; comprised of fructose, citric acid, Streptomycin and Penicillin in double distilled water; pH 7.5) in a ratio of 1 part of semen for 1 part of Tris/Ham F-10 and kept at room temperature in a dark environment.

### Semen Cryopreservation and Thawing

Samples with motility >20%, vigor of ≥2, and 30% normal sperm morphology were subsequently processed for cryopreservation. Depending on the raw sperm concentration, raw semen was added to TEST-yolk buffer-based extender with gentamicin sulfate and 0 or 12% glycerol (Irvine Scientific, USA), in a ratio of 1:1 or 1:2, giving a final glycerol concentration of 6%. Cooled semen was divided into aliquots of 13–29 × 10^6^ sperm/ml. The total number of sperm per straw was adjusted to 10–20 × 10^6^ sperm/straw. Aliquots were then loaded into 0.25-ml plastic straws (100 μl/straw) and sealed with poly-vinyl sealing powder. Semen straws were equilibrated from room temperature to 4°C in a refrigerator for 2 h. A Styrofoam box (inside dimensions 33 × 24 × 23 cm) was filled with liquid nitrogen to a depth of 4 cm. Subsequently, a 1-cm thick Styrofoam “boat” floated on top of the liquid nitrogen for 10 min followed by placing the straws on top of the “boat” for another 10 min (cooling rate of ~220°C/min) before plunging into liquid nitrogen. Straws were then packed into goblet and stored in a liquid nitrogen tank. Thirty straws were frozen from Binturong 1, and 2 from Binturong 1.

Samples from Binturongs 1 and 2 were stored in liquid nitrogen for multiple months before thawing and evaluation. Test straws from Binturongs 1 and 2 were removed from the liquid nitrogen using a long thumb forceps held for 5–10 s in air before being immersed in a water bath (37.5°C) for 5 min. Straws were wiped and emptied into a 2-mL vial (prewarmed at 37.5°C) in a water bath and allowed to stand for 10 min before evaluations for motility, viability, morphology, and acrosome integrity. In addition, plasma membrane integrity was examined using hyperosmotic swelling test (HOS). The semen preparation (2 μl) was incubated at 37°C with 20 μl of HOS solution (made of 0.735 g of sodium citrate and 1.351 g of fructose in 100 ml of distilled water) for 30 min before examining under phase contrast micro-scope (400 × ). A minimum of 200 sperm cells were examined for swollen tail and calculated as a percentage of the total. Sperm motility was then monitored every 5 min for 1 h after thawing.

### Statistical Analysis

Excel analysis (Toolpack package) was used to run data analysis including mean, standard deviation and Student *t*-test. Normal distributions (Kolmogorov-Smirnov-test) and homogeneity of variances (Bartlett's-test) were checked. When data were not normally distributed or variance were not homogeneous, Wilcoxon test were used to compare values. Differences were considered as statistically different when *p*-values were < 0.05.

## Results

### Testicular and Prostate Biometry

**Figure 1:**
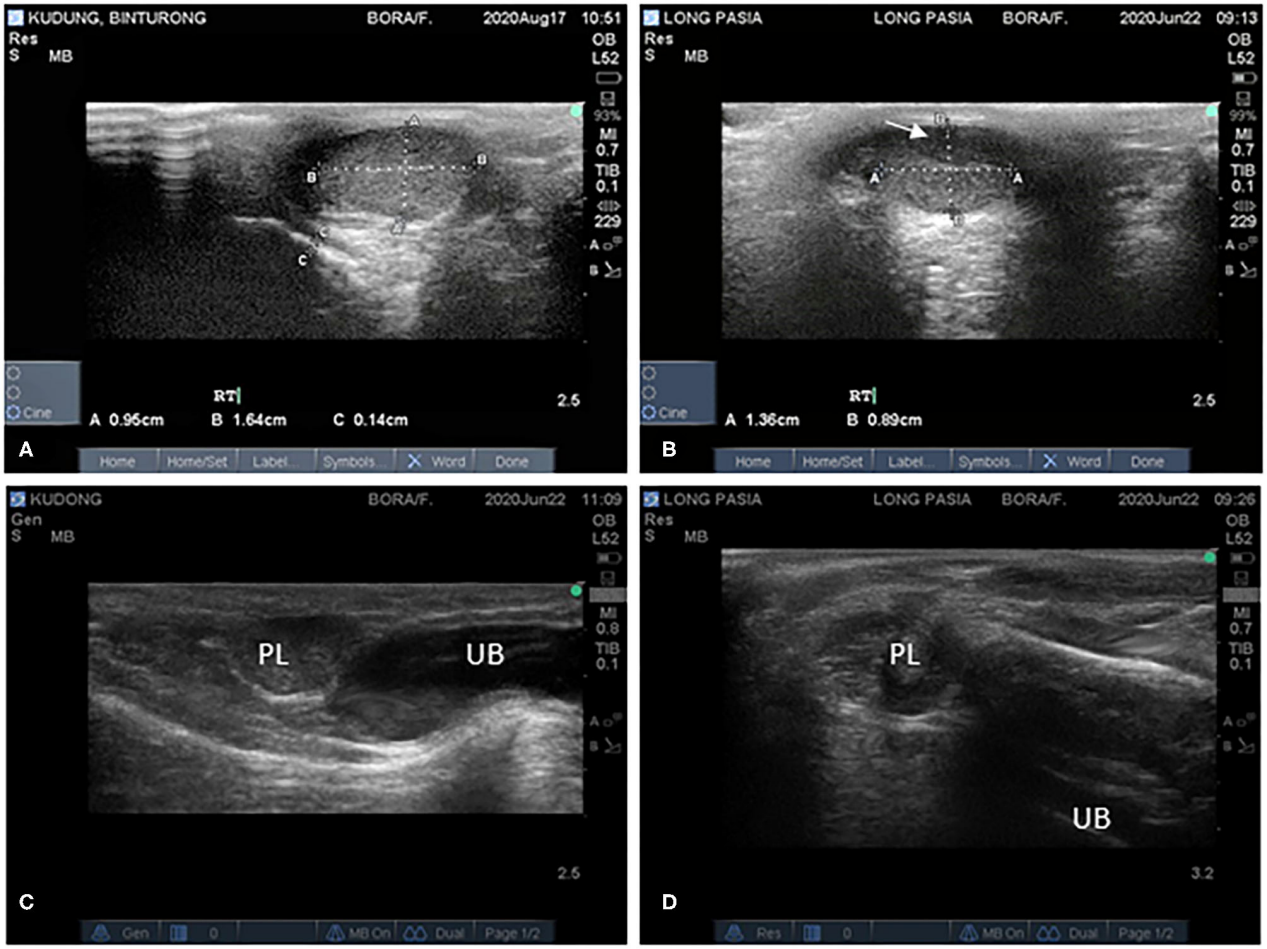
Ultrasonographic images of testes of **(A)** Binturong 1 and **(B)** Binturong 2 (white arrow pointing at hypoechoic structure). Ultrasonographic images of reproductive tracts of **(C)** Binturong 1 (oval-shaped prostatic lobe, PL, with uniform echogenicity) and **(D)** Binturong 2 (heterogenous echotexture of PL). UB, Urine bladder.

Scores of testicular consistencies (ranging from 2 to 4) showed that tissue firmness in both binturongs was comparable. During ultrasonography, testes were well-defined with hyperechoic lines denoting the tunica albuginea at the surface of the testis (Figures 1A,B). Testicles had a homogenous echotexture. However, a hypoechogenic area (1.0 × 0.4 cm) was observed on the right testicle of Binturong 2 (Figure 1B). Total testicular volume was larger (*p* = 0.0326) in Binturong 1 (9 measurements; 3.0 ± 0.6 cm^3^) than in Binturong 2 (5 measurements; 2.3 ± 0.3 cm^3^; Figure 1B).

In Binturong 1, the prostatic lobe was oval-shaped (Figure 1C) with a size of 1.27 × 0.70 cm. It was rounder (1.11 × 1.08 cm) in Binturong 2 (Figure 1D). In Binturong 1, the prostatic lobe was uniformly echogenic with a hyperechoic capsule (Figure 1C). Binturong 2 presented a prostatic lobe with heterogenous echogenicity and hyperechoic areas (Figure 1D).

### Fresh Semen Evaluation

Mean semen pH for the two binturongs was around 7.8 and was not different between the two males (Supplementary Material). Majority of the pH measured in Binturongs 1 and 2 were ≥ 7.0. On four occasions, sample fractions from Binturongs 1 and 2 had lower pH due to urine contamination at the beginning or at the end of the collection (Supplementary Material). Those fractions were discarded. All semen samples from Binturong 1 were good or very good (Supplementary Material). The initial fraction was usually thick, milky or white and became pale white or translucent, to thin, clear or yellowish during collections.

**Figure 2:**
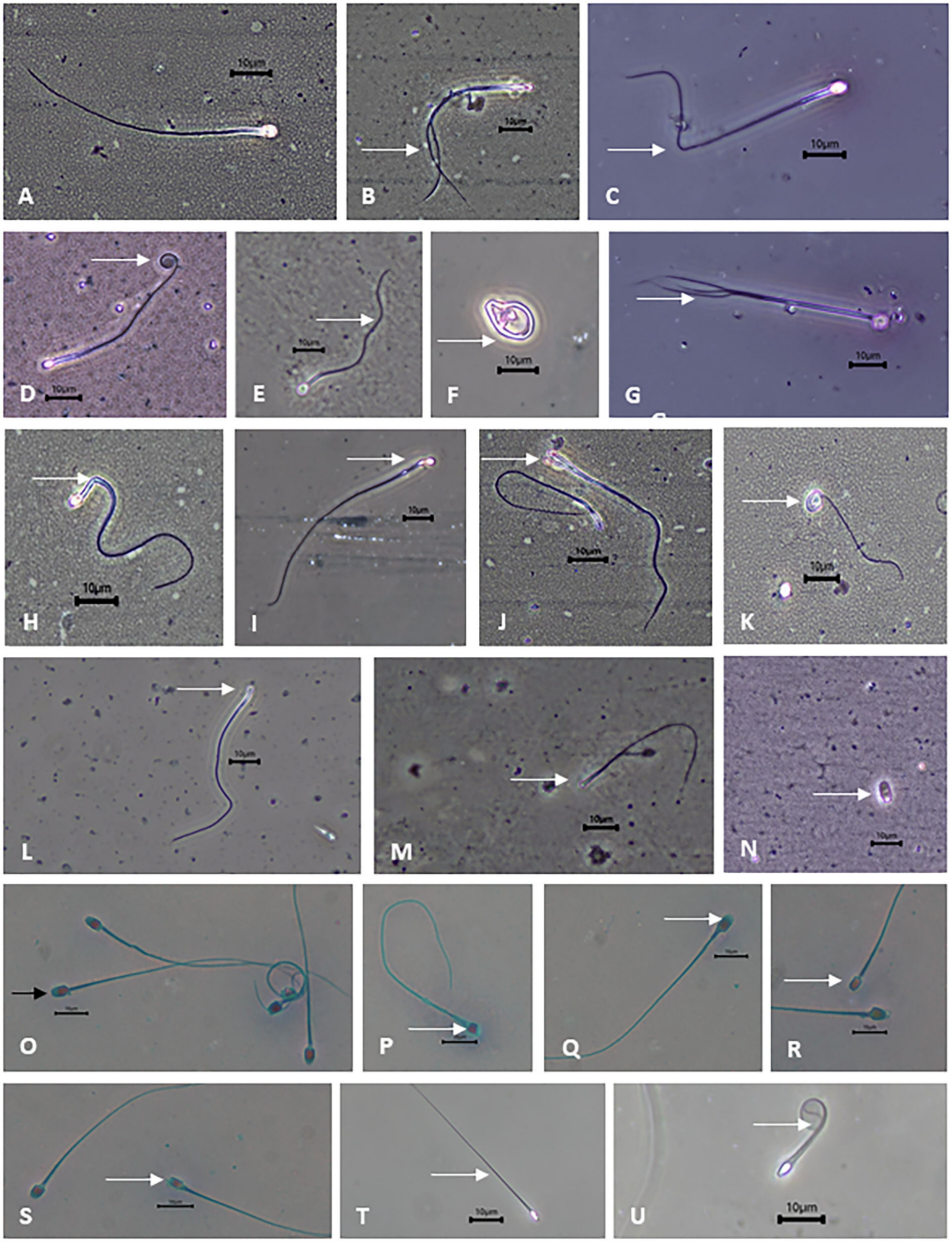
Photomicrographs of binturong spermatozoa under phase contrast microscopy (white arrows are pointing at the defects). **(A)** normal spermatozoa, **(B)** double tail, **(C)** bent tail, **(D)** coiled tail, **(E)** irregular tail, **(F)** tightly coiled tail, **(G)** triple tail, **(H,I)** bent mid-piece, **(J)** bicephalic, **(K)** macrocephalic, **(L)** microcephalic, **(M)** headless, **(N)** detached head. Binturong spermatozoa with **(O)** intact acrosome (black arrow), **(P–S)** non-intact acrosome, **(T)** intact membrane, **(U)** non-intact membrane. Scale bars = 10 μm.

Volume samples were variable across collections in both males (Supplementary Material). Sperm motility, concentration, and viability were higher (*p*-values ranging from 0.0008 to 0.0027) in Binturong 1 compared to Binturong 2 (Table 1). Overall, samples from Binturong 2 were either azoospermic or contained dead sperm cells with very low sperm concentrations and sperm motility index. This resulted in a much lower sperm concentration and total sperm output in that individual. Percentages of normal sperm morphology were similar between Binturongs 1 and 2 (Table 1, Figure 2A).

**Table 1:** Semen characteristics (mean ± SD) of two binturongs.

**Semen characteristic**	**Binturong 1** **(*n* = 11 samples)**	**Range**	**Binturong 2** **(*n* = 5 samples)**	**Range**
pH	7.9 ± 1.1	6.0–9.0	7.7 ± 0.9	6.0–9.0
Volume (μl)	221.0 ± 80.0	50–310	160.0 ± 63.0	60–220
Motility (%)	81.0 ± 10.5^a^	53–90	41.2 ± 28.9^b^	6–70
Progressive motility (%)	69.0 ± 10.8^a^	48–80	17.2 ± 20.9^b^	0–40
Sperm motility index	72.5 ± 10.7^a^	46.5–85	32.6 ± 27.1^b^	3–65
Sperm concentration (×10^6^/ml)	82.0 ± 61.2^a^	8.5–195.0	9.6 ± 13.9^b^	1–34
Total sperm (×10^6^)	18.4 ± 15.5	2.2–36.5	4.5 ± 2.6	2.1–6.1
Viability (%)^*^	71.4 ± 19.1^a^	26.5–88.1	25.1 ± 22.1^b^	11.8–57.9
Normal morphology (%)^**^	28.0 ± 7.9	16.0–42.0	29.4 ± 14.5	17.8–45.6

*In each row, values with different superscripts (a, b) were statistically different (t-test; p < 0.05)*.

* *Viability was assessed only on 9 samples in Binturong 1 and 4 samples in Binturong 2*.

** *Morphology was assessed only on 3 samples in Binturong 2*.

The incidence of sperm abnormalities in both individuals was high (>60%) and comparable (Table 2). In both binturongs, bent-tail was the most common form of abnormalities, followed by coiled-tail, tightly coiled-tail and double-tail (Table 2 and Figures 2B–G). The second highest structural abnormality in both males consisted of mid-piece defects (Table 2 and Figures 2H,I). Defects of the sperm head were not common, and consisted of macrocephalic, microcephalic, abnormal shape and bicephalic (Table 2 and Figures 2J–N). Percentages of acrosome integrity were calculated from last semen collections in 2020 (it was 66.4% from 3 samples of Binturong 1 and 60% from 1 sample of Binturong 2, Figure 2O).

**Table 2:** Morphology of abnormal spermatozoa (mean ± SD) of two binturongs.

**Morphology (%)**	**Binturong 1** **(*n* = 11 samples)**	**Range**	**Binturong 2** **(*n* = 3 samples)**	**Range**
Abnormal sperm	68.4 ± 15.3	29–81	70.6 ± 14.5	54.4–82.2
Bent tail	49.4 ± 11.8	27.4–61.4	39.2 ± 16.7	28.6–58.4
Coiled tail	6.2 ± 8.2	0.0–27.4	15.7 ± 12.7	1.5–26.0
Tightly coiled tail	0.7 ± 1.0	0.0–2.6	0.5 ± 0.9	0–1.5
Double or triple tail	0.1 ± 0.1	0.0–0.5	0.2 ± 0.3	0–0.5
Midpiece defect	12.8 ± 8.8	0.0–29.0	13.6 ± 10.4	6.3–25.5
Macrocephalic	0.7 ± 0.8	0.0–2.0	0.0	0.0
Microcephalic	0.7 ± 1.3	0.0–4.0	1.6 ± 2.5	0.2–4.5
Round head	0.1 ± 0.3	0.0–1.0	0.0	0.0
Bicephalic	0.2 ± 0.4	0.0–1.3	0.3 ± 0.2	0.0–0.5
Narrow head	0.1 ± 0.1	0.0–0.5	0.0	0.0

### Frozen-thawed Semen Evaluation

Samples from Binturongs 1 and 2 were stored in liquid nitrogen for multiple months before thawing and evaluation. Compared to the fresh samples, frozen sperm quality was poor and variable in Binturong 1 (7% motility; 3% progressive motility; 20% of intact acrosomes; 22% of membrane integrity; Figures 2P–U) and of very poor quality in Binturong 2 (no motility and membrane integrity); however, that male had 74% of sperm cells with intact acrosomes. Post-thaw evaluations performed every 5 min for 1 h revealed that initial sperm motility (7%) and viability (18%) of Binturong 1 decreased quickly to 1 and 5.5%, respectively, in the first 15 min. Values then were down to zero 30 min after thawing.

## Discussion

This is the first report about testicular measurements and evaluations of semen characteristics in binturongs based on repeated observations in the same individuals.

The binturong is thought to live between 10 and 15 years in the wild but can reach older ages in captivity (up to 26 years) (1). Males in the present study were not too old and were likely not showing signs of aging. The absence of seasonal effect was consistent with the fact that binturongs can reproduce all year round (1). Because of the small number of individuals involved in the study, interpretation of the data has to be cautious. However, Binturong 1 seemed to consistently provide better samples than Binturong 2. The present study reports the first examinations and measurements of testes and prostate by ultrasonography in binturongs although similar kind of measurements have been reported in other wild carnivores (14). Seasonal changes were not marked like in the closely related mask civet (15). Both binturongs had the same age, but Binturong 2 had smaller testes, which could be due to variation between individuals. Interestingly, Binturong 2 is a proven breeder that produced three offspring in the past. Although some cystic formations were observed in the testicular tissues and prostatic lobes of Binturong 2, the link with the semen quality cannot be established and should be investigated further.

Normal values of fresh semen pH were >7 like in other carnivores (16–18). Similarly, urine contamination could be detected by pH values <7 and were detrimental to the sperm survival. Overall, fresh sperm motility, viability, and acrosome integrity were acceptable and compatible with freezing trials. Sperm concentrations and output also were not different from values observed in wild canids and felids (19, 20). The present findings are the first detailed analysis of semen sample characteristics in binturongs collected repeatedly at different time of the year. Previous data reported by Wemmer et al. (1) were in 3 younger males (18 month old) that had poor semen quality in comparison (20–30% motility, 1–45 million sperm/ml).

Lengths of sperm head (about 4 μm), midpiece (about 9 μm), and tail (about 65 μm) appeared to be within the range of precise measurements previously reported in binturong (11). However, detailed information about sperm abnormalities in binturong was not available until now. The majority of sperm abnormalities observed in both binturongs (tail and midpiece defects) were similar to reports from teratospermic individuals in other carnivore species (12, 21). The high percentages of abnormalities (>60%) is considered as teratospermia and might be due to the high inbreeding level as observed in other wild carnivores (12, 21). Genetic studies are warranted to better understand the origin of teratospermia as well as the differences observed between the two individuals (22).

Overall, testicular measurements did not vary across the different procedures (small standard deviations); however, some semen samples were poor and led to the large standard deviations. Even though semen metrics did not appear to be related to the time of the year, more investigations are warranted to confirm the lack of seasonal variations.

Although based on very few replicates, the results obtained after thawing some sperm samples were not satisfactory. It is difficult to know if there was an individual effect on the poor results and if the semen cryopreservation protocol was suboptimal. Regardless, some spermatozoa could survive the freezing process which is an encouraging indication for future efforts.

While it is challenging to draw conclusions from a small number of individuals, repeated measures at different time of the year provided new information about the testicular size and the semen characteristics in the binturong. Comparison with other Viverrids remains to be done. The present data set will be useful for future research on binturong reproduction and the development of assisted reproductive techniques as well as biobanking of germplasms to sustain small populations.

## Data Availability Statement

The original contributions generated in the study are included in the article/Supplementary Material, further inquiries can be directed to the corresponding author.

## Ethics Statement

The animal study was reviewed and approved by Sabah Wildlife Department [letter with Reference Number JHL (HQ)400-9/82Jld.9(9)].

## Author Contributions

ZZ, SS, and MT worked on the conceptualization. ZZ, MF, and PC wrote the final version. All authors contributed to the collection of information, analysis, and outline of the manuscript.

## Conflict of Interest

The authors declare that the research was conducted in the absence of any commercial or financial relationships that could be construed as a potential conflict of interest.
